# Bufei Decoction Alleviated Bleomycin-Induced Idiopathic Pulmonary Fibrosis in Mice by Anti-Inflammation

**DOI:** 10.1155/2020/7483278

**Published:** 2020-09-08

**Authors:** Shanjun Yang, Wenwen Cui, Mingye Wang, Luming Xing, Yue Wang, Pengyu Zhu, Qisheng Qu, Qiang Tang

**Affiliations:** ^1^Second Affiliated Hospital of Heilongjiang University of Chinese Medicine, Department of Lung Disease, Harbin, China; ^2^Hebei University of Chinese Medicine, College of Integrated Chinese and Western Medicine, Shijiazhuang, China

## Abstract

**Objective:**

This study aimed to investigate the mechanistic action and therapeutic effects of Bufei decoction on idiopathic pulmonary fibrosis (IPF) after inhalation of bleomycin.

**Methods:**

Pulmonary fibrosis model in mice was prepared by atomization inhalation of bleomycin. Then, the mice were randomly divided into five groups (control group, model group, positive group, and treatment group) and administrated the drugs for 4 weeks. H&E and Masson's staining of lung tissues were used to observe the morphological changes and deposition of fibers, and the degree of fibrosis was evaluated by hydroxyproline content. The expression and activation of NF-*κ*B were determined by western blotting and immunohistochemistry. The infiltration of macrophages was detected by immunostaining of CD45 and F4/80 in lung tissues.

**Results:**

In mouse IPF, Bufei decoction alleviated the pathological changes and the deposition of fibrosis by decreasing the content of hydroxyproline of lung tissues. The antipulmonary fibrosis might rely on the effects of preventing the infiltration of inflammatory cells and inhibiting the expression and activation of NF-*κ*B in lung tissue.

**Conclusion:**

Bufei decoction improved the process of pulmonary fibrosis by regulating the activation and expression of the NF-*κ*B signal transduction pathway, which provided a therapeutic option for IPF patients.

## 1. Introduction

Idiopathic pulmonary fibrosis (IPF) is a chronic, progressive, and irreversible interstitial lung disease and progresses to respiratory failure in most cases with a median survival of 3 to 5 years after diagnosis [1]. IPF is complicated in the pathophysiological process whose occurrence and development are still elusive [2]. The typical histopathological features of IPF are patchy injury and proliferation of alveolar epithelial cells, basement membrane exfoliation or injury, alveolar consolidation, and fibroblastic lesions, as well as an abnormal proliferation of mesenchymal cells [3, 4]. The diagnosis criteria for IPF have been proposed with fibrosis of varying degrees [5, 6]. Currently, only two anti-fibrosis agents, Pirfenidone and Nintedanib, have been approved by the FDA [7, 8]. However, the clinical efficiencies are not ideal and the adverse reactions are obvious. Therefore, the clinical specialists frequently combine anti-fibrosis agents with a variety of treatments in practice, including glucocorticoids, anti-inflammatory drugs, antioxidants, immune modulators, and traditional Chinese medicines.

Although the pathogenesis has yet to be fully elucidated, an array of triggers have been found to contribute to IPF, including chemicals, radiation, fibrogenic environmental toxins, or other unknown factors [9]. The inflammatory reactions following those triggers might be the key mechanism for the tissue damage and the accumulation of the extracellular matrix (ECM) proteins, especially proinflammatory cytokines derived from macrophages [10]. At present, a basic consensus about IPF is reached to be a major inflammatory disease due to the growth of inflammatory cells in the lungs [11]. Macrophages as major innate immune cells reside in the lung within alveolar spaces and interstitial tissue. Generally, macrophages have been categorized by function as the classical proinflammatory M1 subtype and the alternative anti-inflammatory M2 phenotype, and both M1 and M2 subtypes are closely associated with the different stages during the disease [12]. M1 macrophages are associated with Th1 immune responses and activated by INF-*γ* and toll-like receptor (TLR) ligands to maximize cytotoxic activity by IL-1*β* and TNF-*α*. The M2 phenotype is associated with tissue repair, angiogenesis, and tissue remodeling, resulting in ECM deposition [13], which means that the polarization of the M2 macrophage subtype is related to the increased severity of pulmonary fibrosis. Moreover, both proinflammatory macrophages and profibrotic macrophages are demonstrated in humans as well as in mouse models [12]. Generally, NF-*κ*B is primarily considered an important regulator of the proinflammatory processes in the M1 macrophages through the production of cytokines such as TNF*α* and IL-6. There is strong evidence that NF-*κ*B activation in subpopulations of macrophages may also represent an anti-inflammatory M2-like phenotype [14]. All these researches suggest that the inhibition of the macrophage activation and NF-*κ*B pathways signaling can be a therapeutic target for the IPF.

Traditional Chinese formulae are under the guidance of the theory of traditional Chinese medicine, usually made up of several herbal medicines. The formulae of Bufei decoction containing *Astragalus membranaceus*, *Polygonum cuspidatum*, *Salvia miltiorrhiza*, *Ligusticum Chuanxiong*, and *Ophiopogon japonicus* have been used for decades in the second afflicted hospital of the Heilongjiang University of Chinese Medicine. Although the possible mechanism of Huogu injection has not yet been thoroughly investigated, some herbs in this formula have been proven to exert therapeutic effects on IPS. *Polygonum cuspidatum* downregulated the level of cytokine (TNF-a) and inhibited the progress of pulmonary fibrosis in rats. *Salvia miltiorrhiza* inhibited or delayed the occurrence and development of bleomycin-induced pulmonary fibrosis by increasing the activity of superoxide dismutase, reducing the content of malondialdehyde and hydroxyproline [15]. We herein examined the effects of Bufei decoction by intervening NF-*κ*B signal transduction pathway in a bleomycin-induced pulmonary fibrosis mouse model [16, 17]. To explore the mechanism of this prescription in the treatment of pulmonary fibrosis and provided a reliable experimental basis for clinical practice, we demonstrated that Bufei decoction could effectively inhibit the infiltration of macrophages and the activity of NF-*κ*B in alveolar macrophages (AM) and reduce the content of hydroxyproline in lung tissue to attenuate the degree of pulmonary fibrosis.

## 2. Materials and Methods

### 2.1. Materials

TCM granules in prescriptions (Jiangyin Tianjiang Pharmaceutical, China). Prednisone acetate (Zhejiang Xianju Pharmaceutical, China). Bleomycin (Solarbio, China). Sodium chloride injection (Harbin Sanlian Pharmaceutical, China). Hydroxyproline Assay Kit (Nanjing Jiancheng Bioengineering, China). RPMI1640 medium (Hyclone, American). Fetal bovine serum (Zhejiang Tianhang Biotechnology, China). Anti-NF-*κ*B antibody (ab16502, 1 : 500). Anti-p-NF-*κ*B antibody (CST3033S, 1 : 500). Anti-Collagen I (ab34710, 1 : 100). Anti-*β*-actin antibody (3700s, 1 : 1000). Anti-CD45 antibody (ab10558, 1 : 1000) and Anti-F4/80 antibody (ab100790, 1 : 100). Anti-rabbit IgG H&L (Alexa Fluor® 488) (ab150077, 1 : 1000). Goat anti-Mouse IgG H&L (IRDye® 800CW) (ab216772, 1 : 5000). Goat Anti-Rabbit IgG H&L (IRDye® 680RD) preadsorbed (ab216777, 1 : 5000). BCA protein assay kit and nucleoprotein extraction reagent (TDY Biotechnology Co., Ltd., China). DAB reagent kit and Rabbit two-step test kit (Zhongshan Jinqiao Biotechnology Co., Ltd., China).

### 2.2. Preparation of Bufei Decoction

Chinese medicine formula granule was composed of 2 g *Salvia miltiorrhiza*, 1.5 g *Astragalus membranaceus*, 1 g *Polygonum cuspidatum*, 2 g *Ligusticum Chuanxiong*, and 3 g *Ophiopogon japonicus*. Before administration, warm water was prepared with a concentration of 0.247 g/ml for immediate use. The daily dose was 4.94 g/kg. The volume of administration is 20 ml/kg.

### 2.3. Animal

A total of 48 male ICR mice, aged 8–12 weeks, weighting 18–22 g, were acclimatized for three days and randomly divided into four groups (*n* = 12 per group): control group (control), bleomycin group (model), bleomycin + prednisone acetate group (positive), and bleomycin + Bufei decoction (treatment). All animals were purchased from the Experimental Animal Center of Liaoning Province.

### 2.4. Establishment of BLM-Induced Pulmonary Fibrosis and Drug Treatment

The pulmonary fibrosis model in mice was prepared by atomization inhalation of bleomycin. When the mice were awake, they were put into a transparent plexiglass box with 30 cm × 30 cm × 20 cm connected with the atomizer and atomized 5 g/L (50%) bleomycin diluent was sprayed into the box through the atomizer tube. Three to four mice were put in the box at a time and exposed to bleomycin for a total of 3 hours and 15 minutes of bleomycin inhalation separated by 7 sessions of 5 minutes of rest. In the control group, mice received saline as a replacement for bleomycin inhalation [4, 18]. On the second day after modeling, all mice except those in the control group and model group were orally treated with saline, and the mice in the positive group and treatment group were continuously administrated with prednisone acetate (at a dose of 0.0064 mg/g) or Bufei decoction (at a dose of 1.235 mg/g) for 4 weeks.

### 2.5. Determination of Hydroxyproline in Lung Tissue

The contents of hydroxyproline were analyzed in lung tissue following the instruction of hydroxyproline assay kit. The pulmonary tissues of mice were ground and homogenized with 1 ml of 6 mol/L potassium chloride solution, hydrolyzed at 95°C for 5 hours, and the pH value was adjusted to 6.0–6.8. According to the instructions, the corresponding reagents were added to the reaction system and mixed thoroughly and then incubated for 15 minutes at 60°C. After cooling, the supernatants were collected after centrifuging at 3500 rpm for 10 minutes. The absorbance value of the supernatant from the samples was measured at 550 nm by a spectrophotometer and calculated for the contents of hydroxyproline on each group.

### 2.6. Histopathological Analysis

Mice were sacrificed and the lungs were harvested on Day 14 and Day 28 after the initial treatment. The lung tissue specimens were fixed in 10% formaldehyde, embedded in paraffin, and cut into 3-5 *μ*m thickness. The lung tissue sections were stained to assess for lung injury and morphological changes using Hematoxylin & Eosin (H&E) and Masson.

### 2.7. Immunohistochemical Staining of Lung Tissue Sections

The tissue sections were dewaxed with xylene and rehydrated through a gradient of ethanol to water. For antigen retrieval, sections were immersed in 0.01 M citrate buffer (pH 6) and heated with a microwave oven. After cooling at room temperature, sections were then transferred into 3% H_2_O_2_ for 15 min to block the endogenous peroxidase activity. After PBS washes, nonspecific antibodies binding to the tissue sections were blocked with 10% normal goat nonimmune serum at 37° for 30 min. After washing off the goat serum, the sections were incubated with primary antibodies (NF-*κ*B and Collagen I) overnight at 4°C. After PBS washes again, sections were rinsed with PBS and incubated with goat antimouse/rabbit secondary antibodies for 15 min at room temperature. After rinsing with PBS, the sections were visualized with diaminobenzidine (DAB) and counterstained with hematoxylin. After sealing with neutral gum, all the sections were photographed under a light microscope.

### 2.8. Immunofluorescence Staining of Lung Tissue Sections for CD45 and F4/80

The tissue sections were dewaxed with xylene and rehydrated through a gradient of ethanol to water. Then, the sections were subjected to fetal bovine serum blocking solution at room temperature for 1-2 h followed by overnight primary antibody incubation at 4°C. Next, the sections were subsequently incubated with the fluorescent secondary antibody for 1 h at room temperature. After counterstaining with DAPI for the nucleus, the sections were sealed with neutral gum, and the fluorescent images were taken by a fluorescence microscope.

### 2.9. Preparation of Bronchoalveolar Lavage Fluid (BALF)

The right lung was perfused with 4°C sterile saline solutions through a 21G needle by the trachea, massaged gently to collect the BALF at Day 28. The collected BALF was centrifuged at 500 ×*g* for 15 min at 4°C. After centrifugation to remove the supernatant, the precipitated cells were washed twice with sterile PBS and cultured in RPM1640 culture medium containing 10% fetal bovine serum and 0.1% penicillin-streptomycin solution at 37°C.

### 2.10. Western Blot for NF-*κ*B and p-NF-*κ*B

The nucleoprotein extracts were prepared from the culture medium of BALF. Briefly, samples were placed in RIPA lysis and extraction buffer containing 0.1% phenylmethanesulfonyl fluoride (PMSF). Tissue protein extracts were then centrifuged and immediately frozen for further western blotting assays. The protein concentration was measured by the BCA kit. Equal amounts of each protein sample were separated on 4%–20% SDS/PAGE gels at 120 V for 1.5 h and transferred on polyvinylidene fluoride (PVDF) membranes at 80–100 V for 1 h. Blots were blocked in a 5% nonfat milk/TBS solution and incubated with the primary antibodies at 4°C overnight. After washing with TBS containing 0.1% Tween 20, fluorescent antibodies were used as secondary antibodies.

### 2.11. Statistical Analysis

The results were presented as mean ± SD. All analyses were performed using SPSS 19.0 statistical software. The statistics and data evaluation were subjected to statistical analysis using one-way ANOVA. ^#^*P* < 0.05, ^##^*P* < 0.01, ^*∗*^*P* < 0.05, and ^*∗∗*^*P* < 0.01 were considered significant.

## 3. Results

### 3.1. Exploration of Pathological Changes in the Lung Tissue

**Figure 1:**
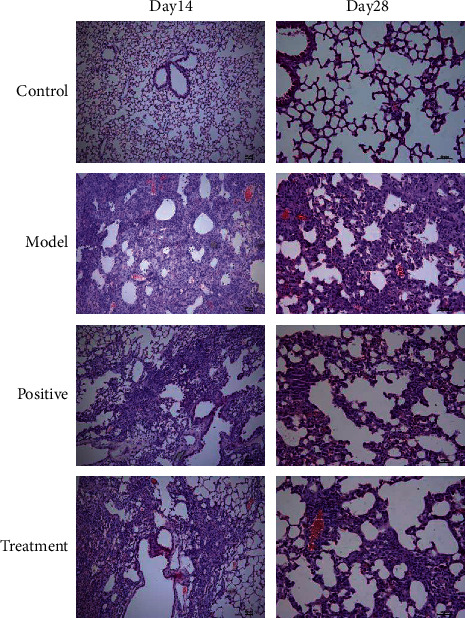
Changes in lung tissue of mice at Day 14 and Day 28 after modeling the pathological structure by HE staining (×100).

To evaluate the effect of Bufei decoction in IPF, H&E staining was performed to observe the pathological changes among groups. As shown in the model group (Figure 1), the BLM-induced IPF at Day 14 and Day 28 were manifested by pulmonary congestion, emphysema of varying degrees, and infiltration of massive inflammatory cells. The degree of pulmonary congestion and emphysema in the treatment groups was less, in addition to a lower level of inflammatory cell infiltration at Day 14 after BLM-induced IPF. After 28 days, the pulmonary inflammation gradually receded, and the inflammatory cell infiltration in the treatment groups was significantly lower than that in the model group; particularly there was no difference between Bufei decoction and prednisone acetate. In the model group, the damaged alveolar structure, liquefaction, necrosis, and local pulmonary fibrosis appeared at Day 14. With the progress of IPF, the main lesion went through alveolar dilatation in the model group at Day 28. In the treatment groups of Bufei decoction and prednisone acetate, the alveolar structure was slightly preserved at Day 14. The pulmonary interstitial hyperplasia was inhibited, but the alveolar dilatation could be observed at Day 28. These results indicated that Bufei decoction could inhibit the inflammatory response and improve the alveolar structure in BLM-induced IPF.

### 3.2. Bufei Decoction Inhibited the Pulmonary Fibrosis Induced with BLM

**Figure 2:**
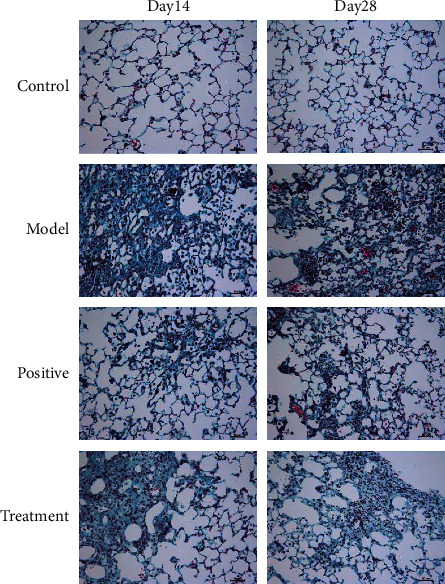
Changes in lung tissue of mice at Day 14 and Day 28 after modeling the pathological structure by Masson staining (×100).

**Figure 3:**
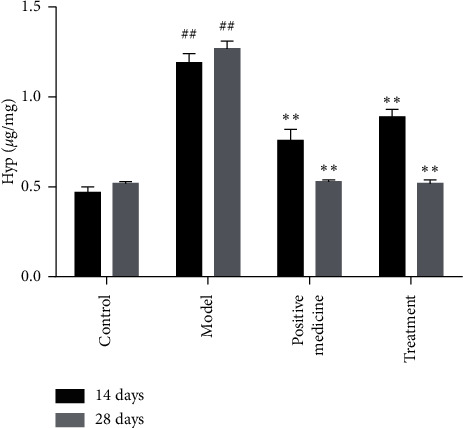
Effect of Bufei decoction on the content of Hyp in pulmonary tissue of mice with pulmonary fibrosis (*n* = 6, mean ± SD). ^##^*P* < 0.01 vs control group. ^*∗∗*^*P* < 0.01 vs model group.

The pulmonary fibrosis is the important manifestation of BLM-induced IPF in mice. To reveal the therapeutic effects of Bufei decoction, we evaluated the extent of pulmonary fibrosis among groups with Masson staining. As shown in Figure 2, the collagen fiber deposition was observed in the lung tissues of the model group at Day 14, mainly concentrated in the terminal bronchial wall and alveolar septum. With the progress of IPF, a large number of collagen fibers dyed blue still deposited in the pulmonary interstitium. In the treatment groups of Bufei decoction and prednisone acetate, a mild extent of pulmonary fibrosis was observed at Day 14 and Day 28. To quantify the extent of pulmonary fibrosis, the hydroxyproline content in lung tissue was measured in each group and is shown in Figure 3. Compared with the control group, inhalation of bleomycin significantly increased the content of hydroxyproline in the lung tissue of the model group at Day 14 and Day 28 (*P* < 0.01). The content of hydroxyproline in lung tissue of the positive group and the treatment group significantly decreased at Day14 and Day 28 (*P* < 0.01).

### 3.3. Bufei Decoction Modulated NF-*κ*B Intranuclear Translocation and Collagen Deposition in BLM-Induced IPF

**Figure 4:**
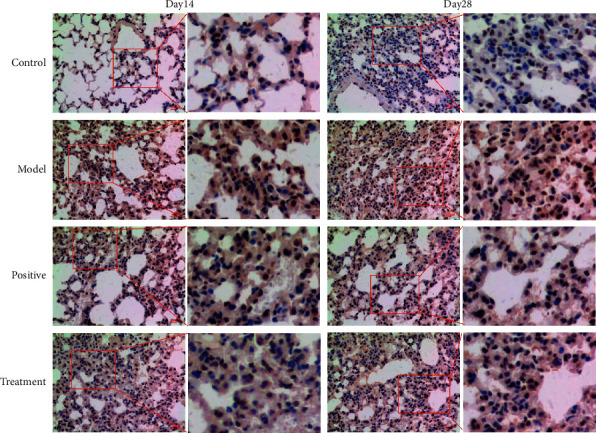
Expression of NF-*κ*B p65 protein in lung tissues of mice in each group (×200).

**Figure 5:**
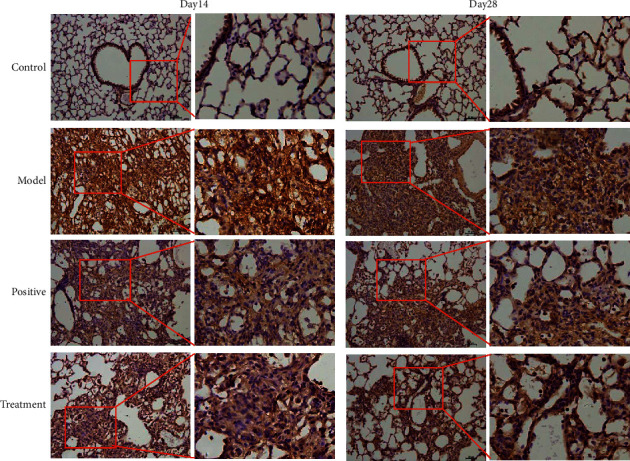
Expression of Collagen I protein in lung tissues of mice in each group (×200).

To further reveal the underlying mechanism of Bufei decoction in BLM-induced IPF, we examined the inflammation-specific NF-*κ*B p65 and fibrotic contributor of type 1 collagen in the lung tissues via the immunohistochemical staining [19]. Compared with the control group, the expression of NF-*κ*B p65 significantly increased and was localized in cytoplasm and nuclei at Day 14 and Day 28 after BLM-induced IPF, especially in the IPF model group. Furthermore, as shown in Figure 4, the expression intensity in the nuclei of the IPF model group was higher than in the nuclei of Bufei decoction treatment group, which meant more NF-*κ*B p65 complexes translocated into the nucleus and played their roles of the nuclear transcription factor to activate the inflammation. Additionally, the expression of type 1 collagen protein was identified as a direct marker during lung fibrosis. Compared with the control group, the expression intensity of type 1 collagen dramatically increased at Day 14 and Day 28 after BLM-induced IPF, as shown in Figure 5. Bufei decoction treatment could inhibit the expression of type 1 collagen and decreased the collagen deposition of lung tissues at Day 14 and Day 28 after BLM-induced IPF.

### 3.4. Bufei Decoction Inhibited the Inflammatory Cell Infiltration

**Figure 6:**
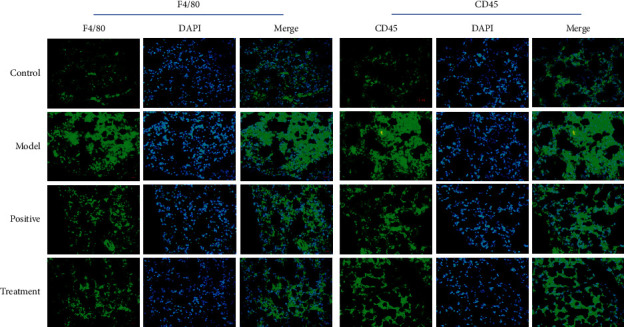
The staining of cell surface markers for leukocytes and macrophages in each group.

The IF staining for the inflammatory cell surface maker of CD45 labelled the infiltrating leukocytes in the lung tissues and the cell surface maker of F4/80 specialized for the infiltrating macrophages. As shown in Figure 6, the results revealed that the infiltration of leukocytes and the subtype of macrophage was significantly increased in the model group. The treatment of Bufei decoction and prednisone acetate reduced the accumulation of inflammatory cells in the lung tissues, which suggested anti-inflammatory effects in the bleomycin-induced lung injuries.

### 3.5. Bufei Decoction Inhibited Expression of NF-*κ*B and p-NF-*κ*B Protein

**Figure 7:**
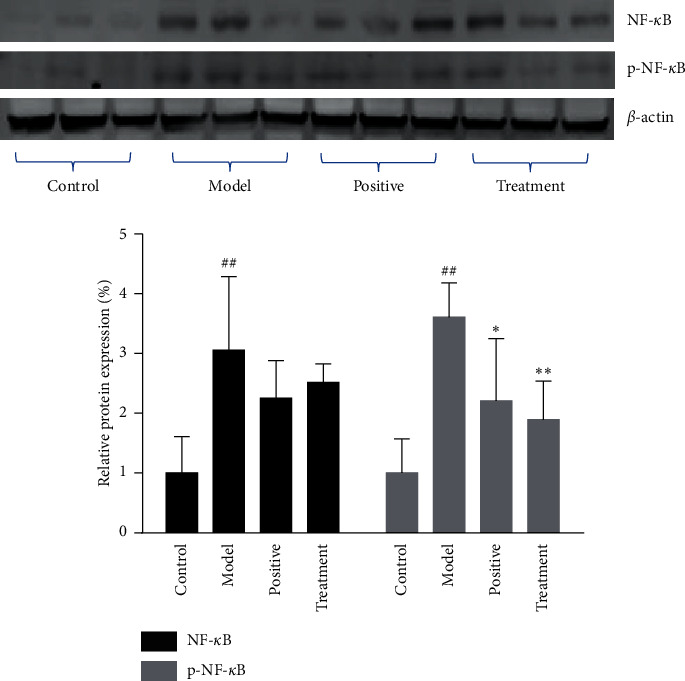
Expression of NF-*κ*B and p-NF-*κ*B protein from the BALF of mice in each group (*n* = 3, mean ± SD). ^##^*P* < 0.01 vs control group. ^*∗*^*P* < 0.05, ^*∗∗*^*P* < 0.01 vs model group.

The western blot for expression of NF-*κ*B and p-NF-*κ*B protein in the BALF of mice at Day 28: as shown in Figure 7, the relative expression of each lane was normalized by the control group in the first three lanes. The results showed that the expression of NF-*κ*B and p-NF-*κ*B protein in the model group was higher than that in the control group, and the positive and treatment groups decreased the expression of NF-*κ*B and p-NF-*κ*B protein at Day 28 after BLM-induced IPF.

## 4. Discussion

IPF has been defined as a type of chronic fibrotic interstitial pneumonia, which is characterized by the progressive and irreversible destruction of pulmonary structures caused by the formation of pulmonary interstitial fibrosis deposition, and ultimately leads to organ dysfunction, gas exchange failure, and respiratory failure [20, 21]. Accumulating evidence shows that IPF is associated with a distinct type of macrophage activation and a comprehensive panel of cytokines produced by the NF-*κ*B signaling pathway. In our clinical practice, the Bufei decoction can moderate the dyspnea symptoms and relieve the progression of acute exacerbation of IPF. Although the clinical application of the Bufei Decoction has achieved satisfactory results, the pharmacological mechanism is still unclear. In the present study, we established a bleomycin-induced pulmonary fibrosis mouse model to demonstrate that Bufei decoction could inhibit the infiltration of macrophages and the activation of NF-*κ*B in lung tissues.

Several studies have proved that the Chinese herbal medicines in Bufei decoction can reduce the degree of pulmonary fibrosis effectively by decreasing the expression of many inflammatory factors in the lung, inhibiting oxidative stress response, and inhibiting the accumulation of extracellular matrix [22–25]. In this study, IPF induced with bleomycin is characterized by increased collagen production and deposition to a varying degree, which will make the alveolar wall thicker and the ventilation function significantly lower, thus leading to the occurrence of pulmonary fibrosis. Hydroxyproline (Hyp) is one of the main components of collagen, and the content of hydroxyproline represents the degree of lung tissue fibrosis. Up to 28 days of oral application, Bufei decoction had a great potential in inhibiting the production of Hyp induced by bleomycin. From the results of H&E staining and Masson staining, Bufei decoction could alleviate the degree of alveolar structural damage and fibroblast proliferation and indeed reduce the content of Hyp, suggesting that it significantly reduced the degree of bleomycin-induced pulmonary fibrosis.

Although the pathogenesis of IPF is not fully understood, there is no doubt that inflammatory injury plays an important role in it. The infiltration and activation of macrophages in the lung tissues play a pivotal role in the IPF. To study whether Bufei decoction could alleviate the inflammatory injury by inhibiting the infiltration of inflammatory cells, the leukocytes and macrophages were detected by immunofluorescent (IF) staining of CD45 and F4/80 in the paraffin section of lung tissue. We revealed that Bufei decoction decreased the number of leukocytes and macrophages, suggesting that Bufei decoction inhibited the inflammatory response in the lung tissues. NF-*κ*B, as one of the main nuclear transcription factors regulating inflammation and immune response, plays an important role in signal transduction in pulmonary fibrosis and other fibrous proliferative diseases by the activation of macrophages [16, 26]. In the process of pulmonary fibrosis, the expression of various cytokines increased in alveolar macrophages by activation of the NF-*κ*B pathway, resulting in excessive fibroblasts proliferation, fibrosis deposition, and pulmonary fibrosis [27–29]. Therefore, the activity of NF-*κ*B in cells directly affects the process of pulmonary fibrosis. Results from the immunohistochemistry showed that Bufei decoction inhibited the staining density of NF-*κ*B and reduced the number of the stained nuclei in the lung tissue sections. The western blotting assay also revealed that Bufei decoction decreased the expression of NF-*κ*B and inhibited its phosphorylation. Given that the macrophages were abundant in the lung tissue of IPF, the expression and phosphorylation of NF-*κ*B in lung tissues might indicate the potential of inactivation of macrophages and played an important role in the occurrence and development of IPF. All the results indicated that Bufei decoction had great potential in inhibiting the inflammatory response in the lung.

To the best of our knowledge, there are no promising treatments in IPF due to the complicated pathogenesis until now. The core principles of formulae are to find an interconnected, complementary, and interdependent relationship for each piece and combine them to yield more beneficial results in treating disease than in using them individually. In this study, we demonstrated that Bufei decoction improved the process of pulmonary fibrosis by regulating the activation and expression of the NF-*κ*B signaling pathway, inhibiting pulmonary inflammation, and alleviating the pathological changes of pulmonary tissue. All these results provided the experimental evidence that Bufei decoction exerted its function by inhibiting inflammatory responses and offered a new therapeutic option for clinicians in the prevention of IPF.

## Data Availability

The data used to support the findings of this study are available from the corresponding author upon request.
